# The NLRP3 inflammasome: a therapeutic target of phytochemicals in treating atherosclerosis (a systematic review)

**DOI:** 10.3389/fimmu.2025.1568722

**Published:** 2025-05-15

**Authors:** Yongchao Liu, Qianyi Wu, Jing Shao, Youmin Mei, Jie Zhang, Qiuyun Xu, Liming Mao

**Affiliations:** ^1^ Department of Immunology, School of Medicine, Nantong University, Nantong, China; ^2^ Department of Periodontology, Nantong Stomatological Hospital, Nantong, China; ^3^ Basic Medical Research Center, School of Medicine, Nantong University, Nantong, China

**Keywords:** atherosclerosis, NLRP3, inflammasome, natural product, herbal compound

## Abstract

Atherosclerosis (AS) is a chronic inflammatory disease characterized by the gradual accumulation of plaques in arterial walls, with its pathogenesis remaining incompletely understood. Recent studies have highlighted that development of AS is closely associated with the aberrant activation of the NLRP3 inflammasome in the arteries. Inhibition of the NLRP3 inflammasome by natural products and formulae derived from Chinese herbal medicines (CHMs) has been shown to alleviate AS-associated pathologies. However, therapies that effectively and safely target the NLRP3 inflammasome remain limited. This review aims to summarize the key discoveries from recent studies on the effects of these natural products and formulae on the NLRP3 inflammasome in the context of AS treatment. A comprehensive literature search was conducted on databases such as PubMed/MEDLINE up to January 2025, yielding 38 eligible studies. Our analysis indicates that certain therapies can effectively prevent arterial inflammation in animal models by targeting multiple pathways and mechanisms related to the NLRP3 inflammasome. This review summarizes the primary findings of these studies, focusing on the therapeutic effects and underlying mechanisms of action. Based on these insights, we propose future strategies to enhance the efficacy, specificity, and safety of existing natural products and formulae for AS treatment. Additionally, this study offers a perspective for future research that may enhance our understanding of the roles and the mechanisms of CHM-derived phytochemicals and formulae in regulating the NLRP3 inflammasome and treating AS.

## 1 Introduction

Atherosclerosis (AS) is a pathological condition characterized by the buildup of fats, cholesterol, and other adhesive substances in the inner lining of an artery, causing arterial wall thickening or hardening. This process progressively obstructs blood flow and contributes to the development of a variety of cardiovascular diseases, including heart attack and stroke (1). Recent epidemiological studies revealed that the prevalence of AS has been continuously increasing and is becoming more prevalent in young adults (2, 3), leading to substantial negative impacts on people’s health and significant social and financial costs (4). High blood cholesterol levels, high blood pressure, and diabetes mellitus are the independent risk factors of AS. These factors may directly activate circulatory monocytes and increase the expression of adhesion molecules and chemokines in endothelial cells, resulting in the recruitment of monocytes, typically, inflammatory Ly6Chi monocytes, to the arterial walls (5, 6). These monocytes transform into macrophages, which acquire a significant amount of lipids and develop into macrophage-derived foam cells during AS (7). Large quantities of inflammatory mediators, including matrix metalloproteinases (MMPs), chemokines, tumor necrosis factor (TNF), interleukin-6 (IL-6), and IL-1, are secreted by foam cells. This exacerbates vascular inflammation and compromises plaque stability (8). Human vascular smooth muscle cells (VSMCs) can dedifferentiate, proliferate, and migrate into arterial plaques through phenotypic regulation (9, 10). AS plaque progression involves VSMCs in the arterial media, which accumulate lipids, transform into myocyte-derived foam cells, and contribute to the formation of fibrous caps (11). These alterations may result in arterial wall thickening and hardening, loss of elasticity, and narrowing of the lumen, resulting in various types of arteriosclerosis, including AS.

Some drugs in the market, such as antiplatelet drugs, anticoagulants, cholesterol-lowering drugs, and blood pressure drugs, have been developed for the treatment of AS. For instance, antiplatelet drugs such as aspirin can reduce the ability of platelets to aggregate and form clots by targeting cyclooxygenase-dependent pathways and inhibit vascular inflammation by suppressing the activity of various inflammatory pathways (12, 13). Anticoagulants exert their effects differently from those of antiplatelet drugs and can inhibit the activities of enzymes in different coagulation cascades or prevent the synthesis of antithrombin in the liver. For example, heparin can inhibit key proteins in the clotting cascade by interacting with antithrombin III (AT) and inducing structural changes and subsequent activation of AT, which inhibits the activity of thrombin and other proteases during coagulation (14). Another type of drugs used in treating AS is the cholesterol-lowering drugs, in which the statins are one of the most used classes of drugs for this purpose. As one of the first-line statins used to treat high-lipid conditions, atorvastatin competitively binds to and inhibits 3-hydroxy-3-methylglutaryl–coenzyme A (HMG-CoA) reductase, thus preventing HMG-CoA conversion to mevalonate (15). Hyperactivation of the mevalonate pathway in endothelial cells is considered a critical causal factor in AS development (16). In addition, Honda et al. showed that antihypertensive drugs, such as angiotensin type 1 receptor blockers (olmesartan) and calcium channel blockers (amlodipine), can benefit hypertensive patients with AS by improving endothelial function and reducing inflammation (17). Although the development and application of these drugs have largely improved AS treatment, they often cause adverse effects. For example, antiplatelet drugs and anticoagulants may increase the risk of bleeding (18). Long-term use of statins may lead to multiple adverse effects such as headache, dizziness, myopathy, and hepatotoxicity (19). Therefore, it is imperative to identify safer and more effective anti-AS agents. Numerous Chinese herbal medicines (CHMs), such as Biejiajian Pill (20), Tongxinluo (TXL) (21), and Qingre Huoxue Decoction (QRHX) (22), and natural products, such as polydatin, have been reported to improve atherosclerotic plaques by inhibiting NOD-like receptor thermal protein domain associated protein 3 (NLRP3) inflammasome activation in basic experiments. Some decoctions, such as Zhibitai (23), and compounds, such as Tanshinone IIA (TSIIA) (24), have been demonstrated to be effective in clinical trials. These studies provide promising information for the development of new therapeutic agents for AS.

The NLRP3 inflammasome is considered a crucial regulator of AS development and a significant modulator of the inflammatory response. Numerous studies have linked inflammasome activation to AS development. NLRP3 triggers pyroptosis and promotes the release of pro-inflammatory cytokines, which increase the risk of atherosclerotic diseases (25). Recent studies also demonstrated that applications of several CHMs may offer new possibilities for treating AS by controlling the NLRP3 inflammasome (26, 27), although there is no available study in the literature to summarize the findings in this field. We therefore conducted an extensive literature search on this topic using a set of carefully selected keywords such as NLRP3 inflammasome, AS, natural compounds, and regulators associated with NLRP3 activation in PubMed database. The details for database searching have been described in the Methods section. After a set of screening steps, a total of 38 publications were selected for inclusion in this study. Our search encompassed *in vitro* and *in vivo* studies, from which we summarized the principal findings reasonably and systematically. This review might offer some valuable perspectives for the development of new drugs for the treatment of AS.

## 2 Methods

**Figure 1:**
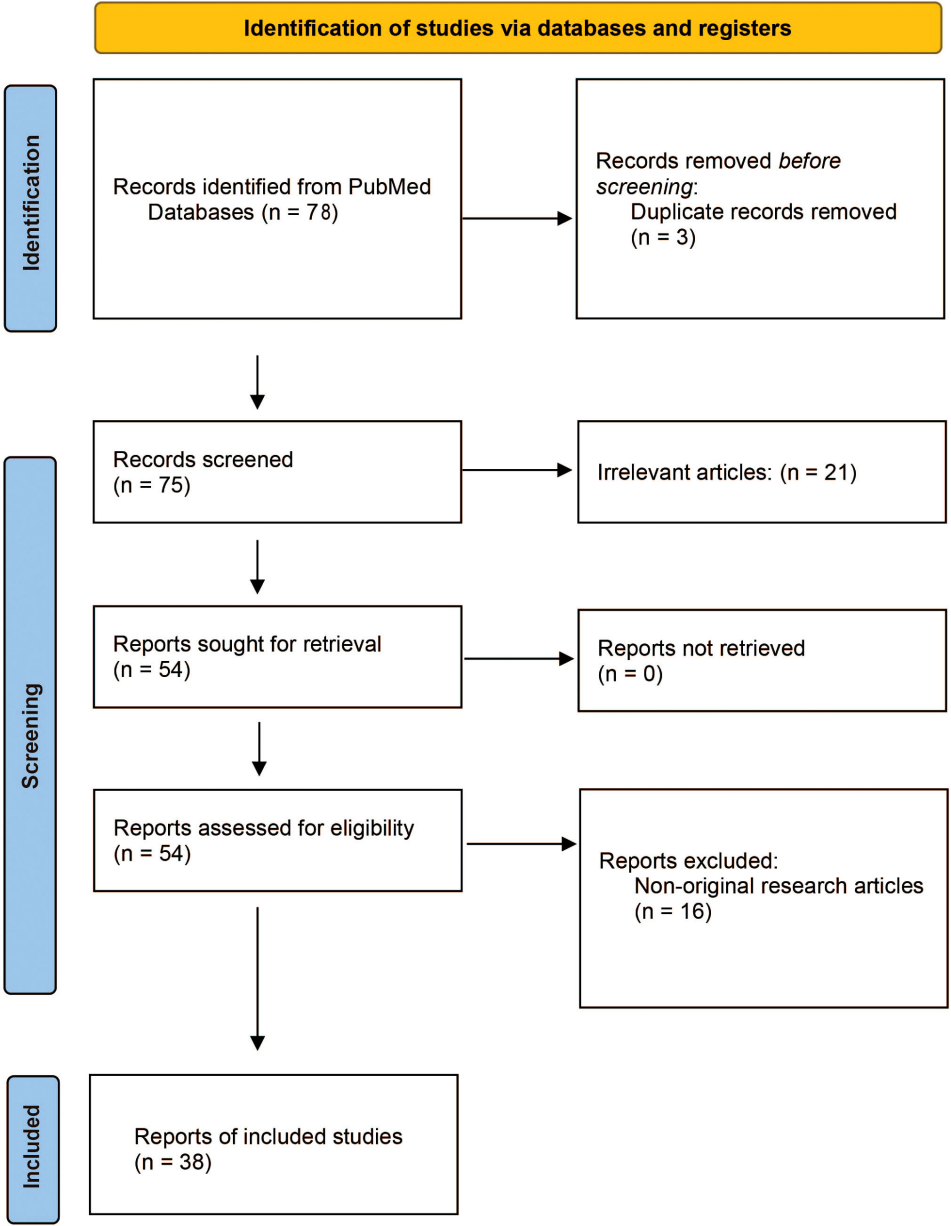
PRISMA flow diagram.

The objective of this systematic review is to explore the impact of traditional Chinese medicine (TCM) and related compounds on NLRP3 inflammasome activity in AS. Following the recommendations of the Preferred Reporting Items for Systematic Reviews and Meta-Analyses (PRISMA) guidelines (28), we conducted a systematic review of primary research articles published from the inception of databases until 30 January 2025 (**Figure 1**).

The systematic search was guided by the “Population, Intervention, Comparison, and Outcome” (PICO) strategy, formulating the research question: How does TCM [Intervention (I)] influence NLRP3 inflammasome activity [Outcome (O)] in AS-related models [Population (P)]? This was assessed by comparing TCM intervention with untreated or placebo control groups [Comparison (C)]. A comprehensive search was performed in the PubMed database using the search query “((NLRP3) AND (Atherosclerosis)) AND (traditional Chinese medicine)” and “((NLRP3) AND (Atherosclerosis)) AND (natural compound).” All authors jointly established and agreed upon the inclusion and exclusion criteria. Studies meeting the following criteria were included: (1) original full-text articles, (2) written in English, (3) focusing on the role of the NLRP3 inflammasome in AS, and (4) investigating the effects of TCM or its active components. Additionally, forward and backward citations of identified studies were reviewed to include further relevant articles. Exclusion criteria included: (1) non-English articles; (2) non-original research articles (such as reviews, medical/conference abstracts, patents, and methodological/tool reports); (3) retracted publications; (4) studies without accessible full manuscripts; and (5) studies not directly related to either NLRP3 inflammasome or TCM. Through the systematic search, 38 eligible studies were identified and included in the review.

## 3 The NLRP3 inflammasome and its role in AS

The primary components of the NLRP3 inflammasome include the sensor protein NLRP3, adaptor protein apoptosis-associated speck-like protein containing a caspase recruitment domain (ASC), and effector protein caspase-1 (29, 30). The activation pathways of the NLRP3 inflammasome mainly include the canonical pathway and the non-canonical pathway. The canonical pathway is the most extensively studied activation mechanism of the NLRP3 inflammasome, typically completed in two steps. The first step is the priming stage, which is mainly triggered by pathogen-associated molecular patterns (PAMPs) or damage-associated molecular patterns (DAMPs) through membrane receptors such as Toll-like receptors (TLRs) or cytokine receptors, activating the nuclear factor kappa B (NF-κB) pathway to induce the transcriptional expression of NLRP3 and pro–IL-1β (31–33). The second step is the activation stage, which is stimulated by various signals, including mitochondrial dysfunction, reactive oxygen species (ROS) production, potassium ion efflux, lysosomal damage, and extracellular ATP (31). Activation signals promote the recruitment of ASC and pro–caspase-1, which leads to the formation of the NLRP3 inflammasome and subsequent cleavage and maturation of pro–IL-1β and pro–IL-18. The other role of caspase-1 is mediating the cleavage of gasdermin D (GSDMD) and releasing the N-terminus of GSDMD to form membrane pores, which trigger the release of intracellular contents and induce pyroptosis (34). Non-canonical pathway is mediated by caspase-4/5 (human) or caspase-11 (mouse), which directly senses intracellular Lipopolysaccharides (LPS) without relying on caspase-1 (35–38). Activated caspase-4/5/11 cleaves GSDMD, forming membrane pores and inducing pyroptosis (39). The subsequent potassium efflux can further activate the canonical NLRP3 inflammasome, amplifying the inflammatory response (40).

**Figure 2:**
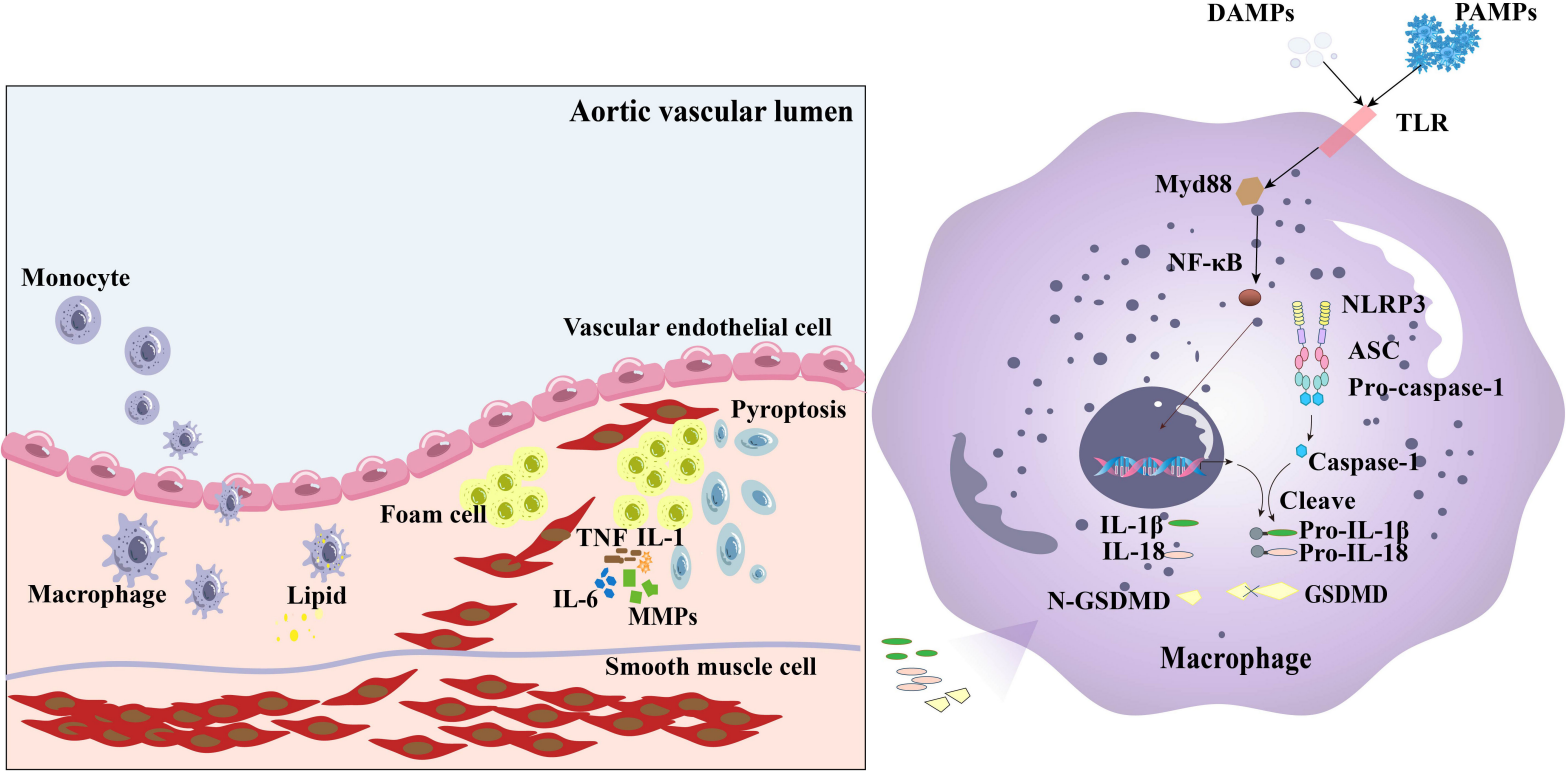
The NLRP3 inflammasome affects the development of AS through various mechanisms. The NLRP3 inflammasome regulates caspase-1 cleavage and activates gasdermin D, leading to pyroptosis. Factors such as adhesion molecules and chemokines in endothelial cells can be released during pyroptosis of the cells, which activate circulatory monocytes and increase the expression of adhesion molecules and chemokines in endothelial cells, leading Ly6C^hi^ monocyte recruitment to the artery wall. These monocytes transform into macrophages, which subsequently develop into foam cells when joined with lipids. Foam cells secrete MMPs, TNF, IL-6, and IL-1, to increase vascular inflammation and decrease plaque stability, leading to arterial wall thickening and hardening, loss of elasticity of the wall, and narrowing of the lumen. VSMCs can dedifferentiate, proliferate, and migrate into arterial plaques, leading to AS plaque progression and myocardial infarction.

As an essential part of innate immunity, the activation of the NLRP3 inflammasome causes arterial inflammation and the development of a plate lipid necrosis by acting as an endogenous danger signal (41). A study conducted by Zhu et al. (42) discovered that NLRP3 gene polymorphisms may affect the risk of ischemic stroke caused by intracranial AS in the Chinese population. Numerous studies have shown that NLRP3, ASC, Caspase-1, IL-1, and IL-18 are highly expressed in the aorta of patients with coronary AS (43–48). Additionally, risk factors for AS, such as total cholesterol (TC), Oxidized low-density lipoprotein (ox-LDL), lipoprotein (a), and coronary stenosis, are all positively correlated with aortic NLRP3 and caspase-1 levels (44, 45). NLRP3 inflammasome–mediated pyroptosis triggers the release of various pro-inflammatory mediators by multiple cells, such as endothelial cells, smooth muscle cells, macrophages, and foam cells, to affect AS progression through a number of mechanisms (49). The released adhesion molecules and chemokines can recruit inflammatory cells to pyroptotic site and initiate the formation of plaques, whereas pyroptotic macrophages can induce plaque instability in patients with advanced AS lesions (50). The contribution of inflammasome to AS progression can also be reflected by the deficiency of inflammasome-related genes. For instance, deficiency of IL-1 or IL-18 has been linked to reduced AS in ApoE−/− mice (51, 52). These findings suggest that NLRP3 inflammasome–related genes play important roles in AS development (**Figure 2**).

## 4 CHMs and natural products affect the progression of AS by regulating the activation of NLRP3 inflammasome

**Figure 3:**
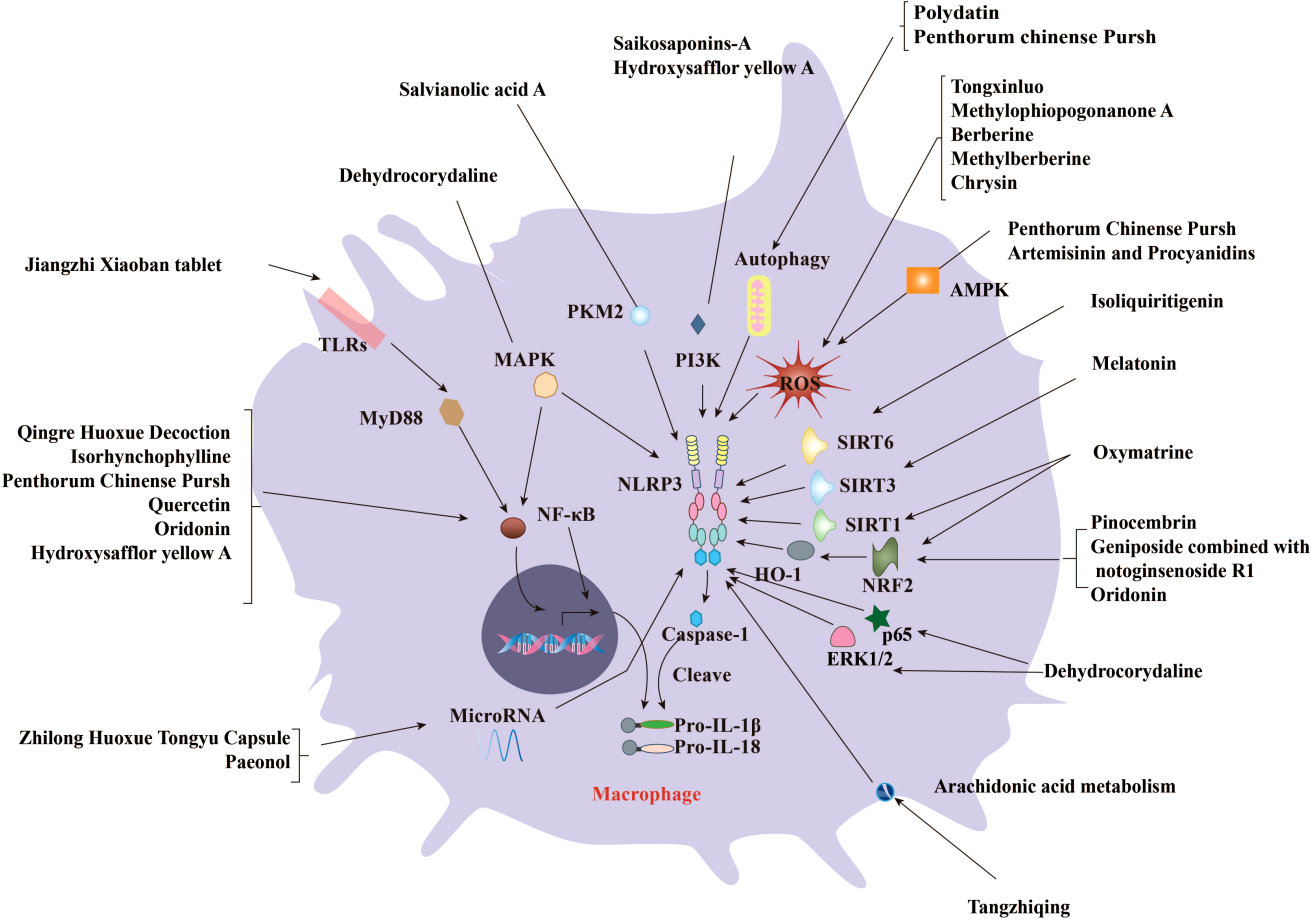
CHMs and natural compounds suppress the progression of AS by inhibiting the activation of NLRP3 inflammasome. Several CHMs and natural compounds suppress AS by regulating various signaling pathways, including the priming of the NLRP3 inflammasome mediated by ROS, NF-κB, and Mitogen-activated protein kinase (MAPK). Meanwhile, many upstream regulators of NLRP3 inflammasome activation such as Nrf2/HO-1, SIRTs, PI3K/AKT, PKM2, microRNAs, gut microflora, and flora-associated metabolites are involved in the anti-AS effects of CHMs and natural products. Several formulae such as Qingre Huoxue Decoction contribute to the inflammatory process of AS development by blocking the NF-κB signaling pathway. Several compounds such as oxytocin prevent AS by enhancing SIRT1/Nrf2 signaling and inhibiting NLRP3 inflammasome. Salvianolic acid A from Danshen can reduce diabetic AS by inhibiting lactic acid–dependent PKR phosphorylation. Saikosaponin-A may affect the activation of NLRP3 inflammasome and alleviate AS by targeting PI3K signaling.

CHMs have been used as an alternative treatment for various diseases, such as diabetes (53), hypertension (54), and asthma (55). Recent studies have demonstrated that certain CHMs or CHM-derived natural products could induce altered activation of the NLRP3 inflammasome and thus affect AS progression. The effect of these medicines on AS is mediated by the regulation of various factors related to NLRP3 activation, such as ROS, NF-κB, nuclear factor E2–related factor 2 (NRF2), sirtuin 1 (SIRT1), phosphoinositide 3-kinase (PI3K)/protein kinase B (AKT), and pyruvate kinase type M2 (PKM2). Here, we provide an overview of these factors affected by traditional medicines (**Table 1**) or natural products (**Table 2**) in affecting AS through regulating the activation of NLRP3 inflammasome (summarized in **Figure 3**).

**Table 1:** CHMs inhibit AS progression by regulating the NLRP3 inflammasome.

Chinese herbal medicine	Prescription	Bioactive ingredient	Target	Mechanism	Disease model	Impact to the host	Toxicity data	References
Tongxinluo	Panax ginseng (*Panax ginseng C. A. Mey.*), leech (*Hirudo nipponica* Whitman.), scorpion (*Buthus martensii Karsch.*), red peony root (*Paeonia veitchii Lynch.*), periostracum cicada (*Cryptotympana pustulata Fabricius.*), eupolyphaga (*Eupolyphaga sinensis Walker.*), centipede (*Scolopendra subspinipes mutilans L. Koch.*), Santalum album (*Santalum album L.*), Dalbergia odorifera (*Dalbergia odorifera T. Chen.*), frankincense (*Boswellia carterii Birdw.*), Semen Ziziphi Spinosae [*Ziziphus jujuba Mill. Var. Spinosa* (Bunge) *Hu ex H. F. Chou.*], and borneol (*Dryobalanops aromatica C.F.Gaertn.*)	Paeoniflorin, Spinosin, Ginsenoside Rg1, Ginsenoside Re, Ginsenoside Rf, Ginsenoside Rb1, Jujuboside A, Ginsenoside Rb2, Ginsenoside Rc, and Ginsenoside Rd	ROS/gut flora	Reduces ROS and inflammatory cytokines; inhibits expression of NLRP3, IL-1β and IL-18; and changes the composition of gut flora	HFD-AS in ApoE−/− mice, and ox-LDL–AS in MAECs	Reduces lipid accumulation and plaque formation	No observed toxicity (0.38/0.75/1.5 g/kg/day for 3 months in mice, and 500 μg/mL for 12 h in MAECs)	(21)
Qingre Huoxue Decoction (QRHX)	*Scutellaria baicalensis Georgi* (Lamiaceae, HQ), *Paeonia veitchii Lynch* (Paeoniaceae, CS), Ligusticum chuanxiong (Apiaceae, CX), *Ilex pubescens Hook. & Arn* (Aquifoliaceae, MDQ), *Carthamus tinctorius L* (Asteraceae, HH), *Dalbergia odorifera T.C.Chen* (Fabaceae, JX), and *Salvia miltiorrhiza Bunge* (Lamiaceae, DS)	Seventy-eight compounds	NF-κB	Decreases the expressions of MCP1, NLRP3, TNFα, iNOS, IL-1β, p-IKK, and p-p65; and increases the expression of CD163, Fizz-1, Ym-1, and Arg-1	HFD-AS in ApoE−/− mice, and LPS-AS in RAW264.7 cell	Reduces weight and the AS plaque area	No observed toxicity (7.5/15/30 g/kg/day for 8 weeks in mice, and 100 μg/mL within 24 h in RAW264.7 cells)	(22)
Penthorum Chinense Pursh	*Penthorum Chinense Pursh*	Four bioactive compounds	AMPK, ROS, and NLRP3	Induces autophagy by activating AMPK pathway to reduce mitochondrial potential and to reduce ROS level	HFD-AS in ApoE−/− mice, and H_2_O_2_-AS in HUVECs	Alleviates the oxidative stress of HUVECs and aortic artery of ApoE-KO mice	No observed toxicity (daily, 0.1 mg/kg/day i.p. for 4 weeks in ApoE-KO mice, and 8 µM within 48 h in HUVECs)	(57)
ZeXieYin formula	Alismatis rhizoma (Zexie, dried tubers of Alisma plantago-aquatica L.), Atractylodis macrocephalae rhizoma (Baizhu, dried rhizomes of Atrac- tylodes macrocephala Koidz.), and Pyrolae herba (Luxiancao, Pyrola calliantha Andres)	Unidentified	NF-κB/NLRP3	Inhibits NF-κB activity and the activation of the NLRP3 inflammasome	HFD-AS in ApoE−/− mice, and LPS-AS in Bone marrow-derived macrophages (BMDMs)	Suppresses HFD-induced AS plaques and plaque rupture	No observed toxicity (daily, 3.8 g/kg p.o. for 8 weeks in ApoE−/− mice, and 200 µM within 24 h in BMDM)	(91)
Jiangzhi Xiaoban tablet	Huzhang (Radix Polygoni Cuspidati), Dahuang (Radix Et Rhizoma Rhei Palmati), Huangqi (Radix Astragali Mongolici), and Danggui (Radix Angelicae Sinensis)	Unidentified	TLR4-NF-κB/NLRP3	Inhibits the expression of TLR4 and suppresses the activaiton of NF-κB and the NLRP3 inflammasome	HFD-AS in ApoE−/− mice	Prevents aortic lesions in AS mice	No observed toxicity (by gavage, 492, 984, and 1,968 mg/kg/day in ApoE−/− mice)	(93)
Zhilong Huoxue Tongyu Capsule	*Astragalus*, *Pheretima*, *Caulis Sargentodoxae*, *Cassia Twig*, and Hirudo	L-Epicatechin, calyx-7-O-,ontodoxaeu Capsulege, 492, 984, and 1,968 mg/kg/day of NF-κB and the NLRP3 inflammasome, and wogonin	miR-30b-5p/NLRP3	Reduces the levels of NLRP3, ASC, caspase-1, IL-1β, and IL-18, miR-30b-5p	HFD-AS in rats, and ox-LDL–AS in HUVECs	Reduces the formation of carotid atherosclerotic plaques and alleviates the pyroptosis of vascular endothelial cells	No observed toxicity (2.52 g/kg/day i.g., for 5 days in rats, and 10% medicated serum 24 h in HUVECs)	(155, 157, 203)
Tangzhiqing	Huangjing (*Polygonatum sibiricum F. Delaroche*), Gouqizi (*Lycium barbarum L.*), Zelan (*Lycopus lucidus Turcz. ex Benth.*), Guijianyu (*Euonymus alatus* (Thunb.) Siebold), and Jiangcan (*Bombyx batryticatus*)	Chlorogenic acid, quercetin, naringenin, luteolin, kaempferol, and baicalein	Arachidonic acid metabolism	Inhibits the expression of TNF-α, IL-6, IL-18, IL-1β, NLRP3, caspase-1, ASC, and GSDMD mRNA	HFD-AS in ApoE−/− mice	Reduces lipid deposition and fibrosis	No observed toxicity (4 or 8 g/kg/day p.o., 42 days in ApoE−/−mice)	(168, 204)

**Table 2:** Natural compounds affect AS progression by regulating the NLRP3 inflammasome.

Compound number	Compound	Compound source	Target	Mechanism	Disease model	Impact to host	Toxicity data	References
(1)	Polydatin	*Reynoutria japonica Houtt*.	Autophagy/NLRP3	Reduces the levels of caspase-1, IL-1β, IL-18, NLRP3, ASC, GSDMD-N, and p62; and increases LC3II/LC3I ratio	HFD-AS in ApoE−/− mice	Improves AS plaques	No observed toxicity (50/100/200 mg/kg/day p.o. for 8 weeks in mice)	(41)
(2)	Methylophiopogonanone A (MO-A)	*Ophiopogonis Radix*	ROS/ NLRP3	Enhances the activities of SOD and CAT to inhibit ROS and NLRP3 inflammasome activation	No *in vivo* data	Inhibits pyroptosis of macrophages	Not available	(69)
(3)(4)	Berberine/ methylberberine	Many plants	ROS/ NLRP3	Inhibits ROS, blocks KLF16-PPARα interaction, and promotes autophagy	Ox-LDL– AS in THP-1, ox-LDL–AS- HUVEC	Improves lipid and glucose metabolism and reduces plaque formation	No observed toxicity (75 µM within 24 h in THP-1; 1 μM 13-MB for 30 h in HUVEC)	(71, 72)
(5)(6)	Artemisinin (ART) and Procyanidins (PC)	Many plants	ROS/NO/ AMPK	Enhances AMPK activity and Inhibits ROS/NO to reduce the activation of NF-κB and NLRP3	HFD-AS in ApoE−/− mice, and ox-LDL–AS- RAW264.7	Suppresses lipid influx and promotes cholesterol efflux	No observed toxicity (5 mg/kg i.v. for 8 weeks in mice, 20 mg/kg i.v. for 1 week in mice, 10 µM PC + 100 µM ART for 24 h in RAW264.7)	(77, 78)
(7)	Chrysin	Many plants	ROS	Inhibits ROS level and NLRP3 expression	Hypercholesterolemia-mediated AS in rats	Increases Nrf2-regulated genes in the aorta	No observed toxicity (100 mg/kg p.o. for 60 days in rats)	(79)
(8)	Paeonol	*Cortex Moutan* root	MiR-223, aminoadipic acid	Decreases the levels of cholesterol and triglycerides, IL-1β and IL-6, NLRP3, ASC, caspase-1, and ICAM-1	HFD-AS in ApoE−/− mice, and plasma derived-exosomes in Rat aortic endothelial cells (RAECs)	Restricts the development of AS and inflammatory reaction	No observed toxicity (200/400 mg/kg p.o. for 6 weeks in ApoE−/− mice)(75/150/300mg/kg p.o. for 4 weeks in rats; 80 µg/ml for 48 h in RAECs; 5, 150, or 300 mg/kg/day p.o. for 4 weeks in mice)	(82, 84, 164)
(9)	Isorhynchophylline	*Rubiaceae uncaria*	NF-κB/NLRP3	Decreases the expressions of NLRP3, NF-кB, caspase-1, and IL-18	HFD-AS in ApoE−/− mice, and LPS-AS in HUVECs or RAW264.7	Inhibits inflammatory response of endothelial cells and macrophages	No observed toxicity (12 mg/L IRN for 24 h in HUVECs or RAW264.7)	(94)
(10)	Dehydrocorydaline (DHC)	*Corydalis yanhusuo*	p65 and ERK1/2	Downregulates CD80, iNOS, NLRP3, IL-1β, and IL-18; inhibits ERK1/2 and p65 activity; and increases LDL-C	HFD-AS in ApoE−/− mice, and LPS and IFNγ-AS in BMDM	Improves aortic stiffness and vascular function	No observed toxicity (5 mg/kg/day i.p. for 12 weeks in mice, and 200 μM DHC for 24 h in BMDM)	(99)
(11)	Quercetin	Various fruits and green vegetable	NF-κB/NLRP3	Inhibits NF-κB activity and the activation of the NLRP3 inflammasome	HFD-AS in Piezo1ΔEC/Ldlr−/−, and ox-LDL–AS in HUVECs	Inhibits the formation of AS plaques	No observed toxicity (50 or 100 mg/kg/day i.p. for 4 weeks in mice, and 6.25/12.5/25 μM for 1.5 h in HUVECs)	(101)
(12)	Pinocembrin	Propolis and honey and other plants such as Populus and Pinus heartwood	Nrf2	Decreases NLRP3, ASC, caspase-1, GSDMD-N, and IL-1β; and upregulates Nrf2, HO-1, and NQO1	HFD-AS in ApoE−/− mice, and ox-LDL–AS in HUVECs	Inhibits AS plaque formation and endothelial cell damage	No observed toxicity (20 mg/kg/day p.o. for 14 weeks in mice, and 5/10/20 µM for 24 h in HUVECs)	(110)
(13)(14)	Geniposide and notoginsenoside R1	*Gardenia jasminoides Ellis* and *Panax notoginseng*	AMPK/ mTOR/ Nrf2	Reduces NLRP3, cleaved caspase-1, IL-1β, and IL-18; inhibits mTOR; and enhances Nrf2 and HO-1	HFD-AS in ApoE−/− mice, and H_2_O_2_-AS in HUVECs	Reduces the aortic plaque and increases collagen fibers	No observed toxicity (50 + 50 mg/kg/day i.p. for 12 weeks in mice, and 400 µM within 24 h in HUVECs)	(112)
(15)	Oridonin	*Isodon rubescens* (Hemsl.) H.Hara	Nrf2/PPARγ/NF-κB/NLRP3	Enhances autophagy and inhibits NLRP3 inflammasome	HFD-AS in rabbits/ApoE−/− mice, and ox-LDL–AS in RAW 264.7 cell	Reduces serum levels of total cholesterol and LDL	No observed toxicity (20 mg/kg, orally, daily, for 8 weeks in rabbits; I.P. injection, 10 and 20 mg/kg, daily for 12 weeks in ApoE−/− mice; and 10 µM in RAW 264.7 cell within 24 h)	(122–124)
(16)	Melatonin	Pineal gland of many organisms	Sirt3/ FoxO3a/ Parkin	Reduces the levels of NLRP3, caspase-1, and IL-1β; elevates the level of Sirt3; and increases deacetylation of FOXO3a and Parkin expression	HFD-AS in ApoE−/− mice, and ox-LDL–AS in RAW 264.7 cell	Reduces the plaque size	No observed toxicity (20 mg/kg/day p.o. for 28 days in mice, 10 and µmol/L for 24 h in RAW264.7)	(129, 131)
(17)	Oxymatrine	*Sophora flavescens*	Sirt1/Nrf2	Suppresses the expression of NLRP3, ASC, cleaved caspase-1, IL-1β, and IL-18 protein	Ox-LDL–AS in HUVECs	Inhibits pyroptosis and alleviates ox-LDL–induced cytotoxicity and apoptosis in HUVECs	Not toxic to HUVECs (8 μM within 1 h in HUVECs)	(133)
(18)	Isoliquiritigenin	Licorice	Sirt6	Reduces the levels of NLRP3, cleaved caspase-1 protein, GSDMD, IL-1β, and SIRT6	TNF-α–AS in HUVECs	Reduces vascular endothelial cell pyroptosis	Not toxic to HUVECs (20 μM within 13 h)	(138)
(19)	Hydroxysafflor yellow A (HSYA)	*Carthamus tinctorius L*	NF-κB/ PI3K/AKT/ xanthine oxidase	Binds to xanthine oxidase and inhibits NLRP3 assembly	HFD-AS in ApoE−/− mice, and ox-LDL or LPS-AS in RAW264.7	Alleviates plaque formation, lymphangiogenesis, and inflammatory mediators	No observed toxicity (6.25, 12.5, and 25 mg/kg, by tail vein injection for 12 weeks, 4 times/week in mice; 150/300 μM within 24 h in RAW264.7; 25/50/100 μM for 15 h in RAW264.7; and 80 μmol/L for 6 h in HUVECs)	(144–146)
(20)	Saikosaponin-A	*Radix Bupleuri*	PI3K/AKT/NF-κB/ NLRP3	Reduces the expression of density lipoprotein receptor-1, CD36, and NLRP3 and the production of pro-inflammatory cytokines	Ox-LDL–AS in THP-1	Reduces lipoprotein uptake and boosts cholesterol efflux	Not available	(143)
(21)	Salvianolic acid A	Danshen	PKM2	Decreases the expression levels of NLRP3, cleaved caspase-1 GSDMD-N, and IL-18; and inhibits the PKR phosphorylation	Streptozocin and the Western diet: diabetes and AS in ApoE−/− mice and glucose in HUVECs	Decreases the atherosclerotic plaque formation	No observed toxicity (10 or 20 mg/kg/day i.p. and p.o. for 4 weeks in mice; and 25 μmol/L for 24 h in HUVECs)	(151)
(14)	Notoginsenoside R1	*Panax notoginseng*	Gut microbiota	Remodels the gut microbiota and inhibits NLRP3 inflammasome	HFD-AS in rats	Improves serum lipid profiles and reduces plaque pathology	No observed toxicity (25 mg/kg by gavage per day for 6 weeks in rats)	(169)

### 4.1 ROS

ROS is oxygen-containing compound with high oxidative characteristics produced during cell metabolism, including superoxide radicals, hydroxyl radicals, and hydrogen peroxide (56). Recent studies have shown that small amounts of ROS can activate autophagy and shield cells from the harmful effects of oxidative stress (57). Excessive concentrations of ROS can directly disrupt the lysosomal membrane, induce the release of pro-degradation enzymes into the cytoplasm, impair autophagy, and induce apoptosis and caspase signaling (58). ROS production is considered to be a frequent upstream mechanism linked to the activation of the NLRP3 inflammasome (30, 59), which results in caspase-1–dependent cell pyroptosis (60). Therefore, it is not surprising that ROS acts as a target of numerous medicines. Indeed, studies have disclosed that many formulae or natural products in CHM can ameliorate AS-related clinical symptoms by targeting ROS, thereby inhibiting NLRP3 inflammasome activation. An example for this type of CHMs is TXL, a Chinese herbal prescription that has been used clinically to treat atherosclerotic cardiovascular diseases (21, 61, 62). Jiang et al. (21) discovered that treatment of TXL could reduce the development of AS in ApoE−/− mice. Using *in vitro* experiments, the researchers demonstrated that TXL could prevent pyroptosis of mouse aortic endothelial cells (AECs) by reducing the production of ROS and its regulatory proteins, Cyclooxygenase-2 (Cox2) and Inducible nitric oxide synthase (iNOS), thereby inhibiting the levels of cleaved-GSDMD, NLRP3, caspase-1, IL-1β, and IL-18. In consistency to the study by Jiang et al., Zhou et al. (63) demonstrated that TXL could reduce the level of ROS because the expression of ROS-induced Nicotinamide Adenine Dinucleotide Phosphate Hydrogen (NADPH) oxidase subunits, including p22phox and gp91phox, was significantly inhibited by TXL treatment. Although this study did not explicitly determine the effect of TXL on the NLRP3 inflammasome, it is plausible to speculate about its involvement based on the existing findings. It is worth noting that Zhou et al. utilized rabbits in their animal models, further substantiating the anti-AS effect of TXL across different animal species. The effective doses are remarkably similar (0.38 g/kg/day in mice in the study by Jiang et al. versus 0.3 g/kg/day in rabbits in the study by Zhou et al.); this consistency reinforces the reliability of the findings. Although inhibiting the activation of the NLRP3 inflammasome is one of the multiple mechanisms by which TXL achieves its anti-AS effect, recent studies have disclosed that TXL could stabilize AS plaque by regulating cell physical function, hormone secretion, and protein binding (61). Moreover, other studies revealed that TXL could affect AS development by changing the gut microflora (64), inhibiting microvascular endothelial cell ferroptosis (65), and blocking miR-155–TNF-α signaling pathway (66). These mechanisms may contribute to the inhibitory effect of TXL on AS development by affecting different cell types or different stages in AS progression. The relative contribution and the interplay of these mechanisms in different cell types or different stages of AS progression should be further investigated in future studies.

Another CHM that could affect AS development by regulating the production of ROS is Penthorum chinense Pursh (PCP) as evidenced in the study by Sun et al. (57). The researchers identified several active components from PCP and demonstrated that these components, especially Thonningianin A (TA), could induce the activation of Ca^2+^/Adenosine 5‘-monophosphate (AMPK)-mediated autophagy, thereby leading to recovery of mitochondrial potential and reduction of ROS level in human umbilical vein endothelial cells (HUVECs). More importantly, ApoE-Knock Out (KO) mice exhibited reduced levels of pro– and cleaved–IL-1β in the aortic artery after administration of TA, demonstrating a reduced activation of the NLRP3 inflammasome. However, the authors did not present data regarding the effect of TA on AS-related symptoms. This is likely due to the use of 2-month-old ApoE-KO mice in the study, which spontaneously develop AS-related symptoms such as fatty streaks 3 months after birth. Therefore, the duration of the study was not sufficient to observe a significant impact of TA on these symptoms. Further experiments may employ older mice to determine this effect. In addition, the specific mechanism by which TA reduces ROS levels and NLRP3 inflammasome activation in HUVECs and ApoE-KO mice remains to be fully elucidated. It is worth noting that a subsequent study by Tian et al. (67) provided further evidence that TA could act as a regulator of the interaction between GPR35 and transient receptor potential channel V4 (TRPV4), a key member of Ca^2+^-permeable channels, to restore endothelial function and vascular tone. This finding highlights the multifaceted role of TA in regulating AS-related biological processes. The relative contribution of TA on GPR35-TRPV4 interaction and Ca^2+^/AMPK-mediated autophagy, as well as their interplay in AS progression, should be further elucidated in ApoE-KO mice and other AS animal models in future studies.

Several natural products derived from CHMs have been shown to have anti-AS roles by modulating ROS level. For instance, *Ophiopogonis Radix* is a CHM frequently used in clinical practice to treat cardiovascular disorders by regulating the stomach, lung, and heart meridians (68). The major homoisoflavonoid extracted from this herbal medicine, methylophiopogonanone A (MO-A) (69), could enhance the activities of superoxide dismutase (SOD) and catalase (CAT), which then reduce ROS buildup and suppress NLRP3 activation and subsequent pyroptosis of macrophages. However, the role of MO-A in macrophages has not been confirmed by other researchers. The potential physiological contribution of MO-A to AS should be extensively evaluated in AS-associated animal models. Another natural product that can affect AS progression through ROS generation is berberine, a chemical found in several plants, such as European barberry. Man et al. (70) reported that berberine can reduce diabetic AS by regulating Krüppel-like factor 16 (KLF16)-peroxisome proliferator activated receptor alpha (PPARα) interaction in ApoE−/− mice. Regarding the mechanisms, Jiang et al. (71) reported that treatment with berberine could block ROS production in ox-LDL–stimulated macrophages and attenuated NLRP3 activation. It should be noted that a derivative of berberine, methylberberine, could promote autophagy and inhibit ROS production and the activation of NLRP3 inflammasome in HUVECs (72). Therefore, targeting ROS may be a common function of berberine derivatives. However, the effective dose of berberine on ROS production (75 μM) was much higher than that of methylberberine (1 μM), indicating that the methyl group could significantly enhance the inhibitory effect of berberine on ROS production. It seems unlikely that berberine and methylberberine target different cell types in AS. Their effects on ROS production and the activation of the NLRP3 inflammasome in various cell types associated with AS should be further determined. Their effects on AS-related symptoms should also be evaluated in animal models such as ApoE−/− mice. Similar to other drugs, the effect of berberine in AS is also multifaceted. Previous studies also reported that this compound could act as an inhibitor of ACLS4 to prevent ferroptosis of endothelial cells (73) and regulate trimethylamine production by targeting the gut microbiota (74). Many other effects of berberine in AS have been extensively discussed in several review studies (75, 76).

The third example of natural products with vasculoprotective effects by regulating ROS production is Artemisinin (ART), a compound extracted from *Artemisia annua* L. Jiang et al. provided evidence that ART treatment could effectively reduce foam cell formation, hyperplasia, and fibrosis in the aortic intima in ApoE−/− mice (77). The mice also exhibited reduced inflammation accompanied by enhanced activation of AMPK pathway and reduced activation of the NF-κB pathway and the NLRP3 inflammasome. Furthermore, Zhou et al. (78) delivered ART and Procyanidins (PC) using biomimetic membrane–coated Prussian blue nanoparticles to treat AS and showed that the nanocomplexes could be scavenger of intracellular ROS and NO and inhibit the activation of NF-κB pathway and NLRP3 inflammasome. It is worth noting that the nanocomplexes could also enhance the activity of the AMPK/mammalian target of rapamycin (mTOR)/autophagy pathway to promote cholesterol efflux. In further studies, to understand the physiological effect of the nanocomplexes, the researchers found that the application of the nanoparticles significantly enhanced the drug concentration in the AS plaque of ApoE−/− mice and thereby ameliorated AS-related symptoms with significantly enhanced efficacy. The significantly higher dose employed in the study by Jiang et al. (50 or 100 mg/kg/day) compared to that in the study by Zhou et al. (5 mg/kg/day) may be attributed to the use of nanoparticles or the distinct drug administration routes (orally administration in the study by Jiang et al. versus intravenous injection in the study by Zhou et al.). However, whether the application of nanoparticles or the differences in drug administration approaches influence the production of ROS and NLRP3 inflammasome activation requires further evaluation in future studies.

As a natural bioflavonoid with various biological properties, Chrysin could reduce ROS level and lipid profile in the intestine and aorta of rats in hypercholesterolemia-induced AS model (79). With the decrease of ROS level, the expression of Nrf2 and its regulated genes were also significantly reduced. Although the researchers did not examine the changes of the NLRP3 inflammasome activation in the rats, the reduction of multiple inflammatory genes, including TLR4, TNFα, NLRP3, and IL17, after treatment of Chrysin indicated a declined activation of the NLRP3 inflammasome. In addition to the inhibitory effect on ROS, Chrysin may affect the progression of AS by regulating the copy number of gut microbial species such as Bacteroidetes and *Lactobacillus*. Moreover, the regulatory role of Chrysin in foam cell formation (80) and endoplasmic reticulum stress–induced apoptosis of the myocardium (81) may also contribute to its atheroprotective effect. However, whether these effects are associated with Chrysin-induced decline of ROS should be further determined in future studies.

An additional example of natural products with atheroprotective effect by regulating ROS is Paeonol, an active compound derived from Paeonia suffruticosa Andr with anti-AS activity. In this line of evidence, Liu et al. (82) showed that oral gavage of Paeonol could significantly reduce AS-related symptoms in an AS model in ApoE−/− mice induced by high-fat diet (HFD). Further analysis of gut microbiota and metabolites revealed that Paeonol could reduce the level of α-hydroxyisobutyric acid (HIBA). The supplementation of HIBA could promote AS inflammation in mice by enhancing ROS levels and activating Thioredoxin Interacting Protein (TXNIP)/NLRP3 pathway and, therefore, induce vascular endothelial cells inflammation. Another mechanism employed by Paeonol to affect AS progression is inhibiting neutrophil extracellular trap–mediated release of citrullinated histone 3 (CitH3) (83), which promotes the activation of the NLRP3 inflammasome and thereby regulates foam cell formation in ApoE−/− mice. It is worth mentioning that the inhibitory effect of Paeonol on AS could also be observed in Sprague-Dawley (SD) rats treated with HFD. Shi et al. (84) found that a similar dose of Paeonol (75–300 mg/kg/day), as used in mice (200–400 mg/kg/day) via oral gavage, could effectively reduce TC, triglycerides, and inflammatory cytokines such as IL-6 and IL-1β in the rat’s serum. Additionally, it could significantly enhance the survival of AECs by upregulating miR-223, which, in turn, inhibits NLRP3 inflammasome activation. Although it remains to be determined, whether the generation of miR-223 and CitH3 concurrently occurs in the same animal model, as well as their influence on the generation of ROS, warrants further investigation in future studies.

Additionally, a synthetic compound derived from the natural product phlorizin, dapagliflozin [an inhibitor of Sodium-glucose co-transporter 2 (SGLT2)], was also shown to affect the progression of AS by regulating ROS production. A study by Leng et al. (85) reported that dapagliflozin treatment ameliorated aortic AS formation in streptozotocin-induced diabetic ApoE−/− mice. The compound also inhibited macrophage infiltration and improved lesion stability. On molecular level, dapagliflozin reduced the levels of mitochondrial ROS, IL-1β, IL-18, and NLRP3 in aortic tissues. The findings of Leng et al. were subsequently confirmed by Hodrea et al., showing that dapagliflozin could prevent thickening of aortic intima-media and cardiac inflammation of streptozotocin-treated SD rats. However, a study by Taberner-Cortés et al. (86) challenged the effect of dapagliflozin on AS progression. The researchers explored the effect of dapagliflozin on AS in ApoE−/−Irs2+/− mice, which have partial inactivation of the insulin signaling. Their findings showed that treatment of dapagliflozin did not affect body weight, plasma glucose, or lipid induced by high-fat and high-cholesterol diet in the mice. This discrepancy may be associated with the partial deficit of the insulin signaling. The interplay between dapagliflozin-triggered genes and the insulin signaling should be extensively investigated in future, which may be helpful to clarify the exact contribution of dapagliflozin in AS.

### 4.2 NF-κB/MAPK

NF-κB is an essential transcription factor involved in the regulation of numerous biological processes in nearly all animal cells. It is crucial in cellular reactions to external stimuli such as environmental factors, cellular stress, and hypoxia, thus affecting many AS-associated biological processes, including the transcription of NLRP3 inflammasome–related genes (60, 87). A recent study showed that the herbal remedy QRHX may prevent and cure arteriosclerotic cardiovascular disease (ASCVD) by blocking NF-κB (22). Regarding its effect on AS, Jin et al. (88) showed that QRHX can promote macrophage M2 polarization and decrease the level of IL-1β, MCP1, NLRP3, and TNF-α in serum by blocking the NF-κB signaling pathway, which was concomitant with a decreased spread of AS. A subsequent study of the same group of researchers provided evidence that the inhibitory effect of QRHX on NF-κB signaling pathway was modulated by miR-26a-5p from macrophage-derived exosomes (89). Therefore, the inhibitory effect of QRHX on the development of AS was achieved by enhancing miR-26a-5p and thereby blocking the NF-κB signaling pathway, which suppresses NLRP3 inflammasome activation and macrophage polarization. It has been reported that QRHX could ameliorate myocardial ischemia–reperfusion injury by regulating autophagy-endoplasmic reticulum (90). This new mechanism of QRHX involved in regulating AS and interplay with NLRP3 inflammasome activation, as well as the generation of particular regulatory microRNAs, should be explored in future studies.

A study by Huang et al. (91) showed that the NF-κB/MAPK pathway is also the target of ZeXieYin formula (ZXYF) in alleviating AS in ApoE−/− mice. ZXYF is a traditional herbal medicine used in relieving spleen dampness and heat accumulation syndrome. ZXYF treatment could significantly suppress HFD-induced AS plaques and plaque rupture by inhibiting NF-κB/MAPK signaling pathway, which restrained the activation of the NLRP3 inflammasome and promoted the differentiation of M2 macrophages. In this process, ZXYF-induced inhibition of MMPs also played a significant role in stabilizing plaques. Related studies from the same research group demonstrated that ZXYF could reduce the level of trimethylamine oxide (TMAO) in serum of AS mice, which is a metabolite of gut microbiota and may have possible adverse effects on cognitive functions. To explore this possibility, the researchers established TMAO-induced cognitive impairment model in mice and found that ZXYF treatment could significantly ameliorate TMAO-induced cognitive defects such as the learning and memory ability (92). Their findings provided further evidence that ZXYF is an effective and safe drug for AS. Exploring the possible interaction between TMAO and the activation of NF-κB and NLRP3 inflammasome may further enhance our understanding of therapeutic roles of ZXYF in treating AS. The third formula that affects progression of AS by regulating NF-κB and NLRP3 activation is the Jiangzhi Xiaoban tablet (JZXB) (93). It could suppress the levels of triglyceride, TC, and low-density lipoprotein cholesterol (LDL-C) while enhancing the level of high-density lipoprotein cholesterol (HDL-C) in serum and the pathological changes of aorta in AS mice. Meanwhile, JZXB inhibited the activation of NF-κB and the expression of NLRP3 and IL-1β. To date, only one study has been conducted to explore the effects of JZXB in AS. The findings require further evaluation by other researchers, and the related mechanisms warrant an extensive investigation.

Isorhynchophylline (IRN) is a tetracyclic indole oxide alkaloid commonly found in the plant *Rubiaceae uncaria.* A recent study has reported that it may prevent progression of AS by inhibiting NF-κB (94). IRN has a wide range of pharmacological activities, including neuroprotection, proliferative inhibition, anti-inflammatory, and antihypertensive activities (95–97). Wang et al. (94) discovered that IRN can reduce the inflammatory response of endothelial cells and macrophages by decreasing the levels of NLRP3, IL-18, and caspase-1 in aorta of ApoE−/− mice and thus reducing plaque formation. To further explore the detailed mechanisms by which IRN regulates AS progression, a subsequent study conducted by another research group conducted Liquid Chromatography-Mass Spectrometry (LC-MS)–based metabolomics analysis on serum samples from ApoE−/− mice. Their findings identified three metabolic pathways, namely, glycerophospholipid metabolism, linoleic acid metabolism, and alpha-linolenic acid metabolism, which were significantly disturbed by IRN treatment (98). These pathways may serve as critical focal points for future mechanistic studies. Accordingly, the potential interactions between these metabolic pathways and the NF-κB/NLRP3 pathway merit further evaluation.

Dehydrocorydaline (DHC), an alkaloid derived from *Corydalis yanhusuo*, was shown to alleviate AS in the study by Wen et al. (99), demonstrating that DHC inhibited the development of HFD-induced AS and decreased the levels of CD80, iNOS, NLRP3, IL-1β, and IL-18. Their subsequent mechanistic studies revealed that DHC reduced AS in ApoE−/− mice by reducing Extracellular-regulated kinase 1/2 (ERK1/2)- and p65-mediated pathways in macrophages. To further determine the potential effects of DHC on the functions of VSMCs, the researchers found that DHC-treated VSMCs exhibited increased expression of VSMC contractile phonotype markers such as calponin 1 and smooth muscle 22α. Moreover, DHC could suppress the proliferation and migration of VSMCs (100). Therefore, the effects of DHC in ameliorating AS are achieved by regulating various cell types involved in the progression of AS. DHC-regulated NF-κB/NLRP3 pathway that plays a role in the function of VSMCs merits further investigation. The identification of the binding partners of DHC in macrophages may offer novel insights into the mechanistic studies of this compound in treating AS.

Quercetin is a natural antioxidant with potent anti-AS effect. The effect of quercetin in treating AS is achieved by regulating various pathways, in which one of the most important is the NLRP3 inflammasome. Wang et al. (101) demonstrated that quercetin could remarkably suppress the formation of AS plaques and inhibit the activation of the NF-κB activity and subsequent NLRP3 activation in endothelial cells triggered by exposure to ox-LDL or mechanical stretch. In studies exploring the mechanisms, the researchers found that quercetin can modulate the activation of Piezo 1 channels as evidenced by altered Yoda1-evoked calcium response in HUVECs. Genetic depletion of Piezo1 in vascular endothelial cells substantially compromised quercetin’s effect on NF-κB and NLRP3, further demonstrating the essential role of Piezo1 for quercetin to achieve its anti-AS effects. In a related study, Li et al. (102) confirmed the anti-AS effect of quercetin using HFD-treated ApoE−/− mice. In their studies, in addition to showing that quercetin could alleviate atherosclerotic lesions and reduce lipid retention, the researchers conducted proteomic analysis and found that quercetin could inhibit Gal-3, whose expression could be enhanced by HFD. Their further analysis disclosed that Gal-3 had correlated expression with NLRP3 and could bind to NLRP3 and promote NLRP3 inflammasome activation. Thus, the inhibition of Gal-3 may also contribute to the inhibitory effect of quercetin on NLRP3 inflammasome. Further studies may clarify the potential interactions between Gal-3 and Piezo1, as well as their relative contribution to the activation of NLRP3 inflammasome and AS progression.

Another compound that has anti-AS effect through regulating NF-κB and subsequent NLRP3 expression is polydatin, a bioactive component derived from the roots of *Reynoutria japonica* Houtt. In this line of evidence, Zhang et al. (41) treated ApoE−/− mice with polydatin and found that the formation of AS plaques, and the levels of lipid induced by HFD could be prevented by polydatin administration. Notably, polydatin inhibited NLRP3 expression, suppressed pyroptosis, and promoted autophagy of macrophages. The effect of polydatin has not been studied by other researchers. Its effect and the related mechanism require further investigation. For instance, identifying the precise binding molecules of polydatin and elucidating how these molecules regulate autophagy and the activation of the NLRP3 inflammasome could enhance our understanding of polydatin’s anti-AS effects. Advances in these studies may facilitate the development of novel polydatin-based anti-AS drugs.

### 4.3 Nuclear factor E2–related factor 2/heme oxygenase-1

Nrf2 is a cytoprotective and redox-sensitive transcription factor that is widely expressed in various cells and tissues and is a major regulator of anti-oxidative and cytoprotective genes (103). The Nrf2 signaling pathway is tightly linked to the development of AS, possibly by modulating the host response to oxidative stress (104). Nrf2, together with its downstream partner heme oxygenase-1 (HO-1), can inhibit cell apoptosis and AS-related inflammatory response (105). Pinocembrin (PIN), a natural flavonoid present in propolis, honey, and some other plants, exhibits various biological activities and has anti-AS effects (106–110). Wang et al. (110) showed that PIN could reduce the expression levels of NLRP3, ASC, caspase-1, GSDMD-N, pro–IL-1β, and IL-1β, and, meanwhile, upregulate the activation of the Nrf2/HO-1 axis. This effect led to a protective role of PIN on ox-LDL–stimulated HUVECs against pyroptosis caused by the NLRP3 inflammasome and subsequent GSDMD cleavage. Despite the current literature only reporting the effects of PIN on endothelial cells in AS, its inhibition on PGE2 production in LPS-stimulated RAW 264.7 and U937 cells indicates a potential effect on macrophages (111). The impacts of PIN on macrophage function or other cell types involved in AS progression require further investigation. Additionally, the binding partners of PIN upstream of NRF2 and NLRP3 inflammasome activation warrant further identification.

The combination of Geniposide (GP) and Notoginsenoside R1 (NR) has been studied in AS-related models. GP is an iridoid glycoside and is the primary active ingredient obtained from the dried fruit of *Gardenia jasminoides Ellis* (112). It may reduce AS development through regulating inflammation, lipid metabolism, macrophage activation, and autophagy (113–116). Owing to the remarkable efficacy in controlling both internal and external hemorrhages, promoting hemostasis, and lowering blood pressure, NR has been clinically utilized in China for the treatment cardiovascular and cerebrovascular diseases (117, 118). A study by Liu et al. (112) investigated the effect of GP combined with NR (GN combination) in AS progression and found that GN could alleviate AS-related symptoms and reduce the levels of NLRP3, cleaved caspase-1, and IL-1β and IL-18 in aortic of the ApoE−/− mice. Using *in vitro* and *in vivo* studies, the GN combination was shown to activate AMPK and inhibit mTOR, which enhanced Nrf2 and HO-1 levels. However, the precise binding partners of GN and their potential interactions with Nrf2 and HO-1 have not been determined in AS inhibition, requiring further investigation. It should be noted that the single application of GP was reported to inhibit foam cell formation and lower reverse lipid transport by regulating p38/MAPK signaling pathway (119). Thus, the clarification of the relative contributions of both GP and NR may be helpful for further improvement of the efficacy and lowering the toxicity of the combination. Moreover, recent studies indicated other mechanisms by which GP alleviates AS development, such as regulating miR-101/MKP/p38 pathway (113) and macrophage polarization mediated by perivascular adipocyte-derived CXCL14 (120). This evidence further enhanced the complexity of the mechanisms of this compound in modulating AS. Unraveling these complex interactions will pave the way for more effective therapeutic interventions against AS using GP- and NR-based drugs.

Oridonin is herbal compound derived from Isodon rubescens (Hemsl.) H. Hara with multiple biological activities (121). The role of Oridonin in AS was initially reported by Sultan et al. (122). It could reduce serum levels of TC and LDL by inducing autophagy and thus inhibiting the transcription of NLRP3 in rabbits. In subsequent studies, Wang et al. (123) found that the inhibitory role of oridonin on AS was mediated by upregulating the activity of Nrf2 and thereby inhibiting the activation of NLRP3 inflammasome in ApoE−/− mice. Another mechanism of oridonin affecting AS progression was reported by Zhang et al., showing that oridonin could inhibit foam macrophage formation by regulating FABP4/PPARγ signaling pathway and thereby inhibiting NF-κB translocation in ApoE−/− mice (124). It is noteworthy that the effective dose of oridonin used in the study by Zhang et al. (5 mg/kg/day) was significantly lower than that used in the study by Wang et al. (10 and 20 mg/kg/day). Given that the drug purity and administration method were identical in both studies, this variation may be attributed to the difference in the age of mice used (6 weeks old in the study by Zhang et al. versus 8 weeks old in the study by Wang et al.). The two aforementioned mechanisms should be further evaluated in animal models of the same age. Identifying the cellular targets of oridonin could further enhance our understanding of its mechanisms in regulating NLRP3 inflammasome activation and AS progression.

### 4.4 Sirtuins

SIRT signaling blocks NLRP3 activation in vascular endothelial cells (125). Numerous studies have shown that the histone deacetylase SIRT family (SIRT1-7) is crucial in AS pathophysiology (126, 127). The Nrf2 signaling pathway can be activated by SIRT1, and it has been demonstrated that stimulation of the Nrf2 pathway reduces endothelial cell damage, suppresses inflammation, and has anti-atherosclerotic properties (104, 128). A growing number of studies have suggested that boosting SIRT/Nrf2 signaling may diminish activation of the NLRP3 inflammasome and pyroptosis (129, 130). Ma et al. (131) showed that melatonin, a natural product produced by the pineal glands of several organisms, can ameliorate AS plaque size and vulnerability in ApoE−/− mice. Moreover, melatonin could reduce NLRP3 activation and the subsequent IL-1β production within AS lesions by enhancing the level of SIRT3 in macrophages, which induced activation of FOXO3a and Parkin to regulate mitophagy. Zhao et al. (132) further demonstrated the efficacy of melatonin in alleviating AS and provided evidence that melatonin can mitigate cigarette smoke-induced AS-like symptoms in SD rats by activating the Nrf2 pathway and inhibiting NLRP3 inflammasome activation in endothelial cells. Although there are differences in animals species, SIRT3 or its family members may similarly function as targets of melatonin for treating AS, albeit with variable effective doses (10 mg/kg/day in SD rats versus 20 mg/kg/day in ApoE−/− mice). Future studies should clarify this issue and investigate the potential interactions between SIRT3 and Nrf2 in SD rats exposed to cigarette smoke. Thus, melatonin holds promise as an inhibitor of the NLRP3 inflammasome in AS by targeting multiple signaling pathways in both macrophages and endothelial cells.

Oxymatrine is a quinolizidine alkaloid discovered in Chinese herb *Sophora flavescens* Aiton (133). It has several pharmacological properties, including anti-inflammatory, antioxidant, antifibrotic, and cardiovascular protective activities (134, 135). Regarding its impact on AS, a study by Jin et al. (133) provided evidence that oxymatrine can mitigate ox-LDL–induced injury of HUVECs by suppressing the expression of NLRP3, ASC, cleaved caspase-1, IL-1β, and IL-18. Further analysis revealed additional mechanisms such as promoting the nuclear translocation of Nrf2 and enhancing HO-1 levels. This effect was mediated by SIRT1 because si-SIRT1 transfection decreased the anti-inflammatory activity of oxymatrine. The effect of oxymatrine on AS-related biological processes was previously demonstrated by Zhang et al. (134), showing that the compound could inhibit homocysteine-induced autophagy of HUVECs by regulating macrophage migration inhibitory factor–mTOR signaling pathway. Whether oxymatrine-mediated activation of the SIRT1 and Nrf2/HO-1 signaling pathways plays a role in the occurrence of autophagy and their potential contributions to the activation of the NLRP3 inflammasome requires further investigation. Similar to other natural compounds, oxymatrine may modulate the function of various cell types implicated in the pathogenesis of AS by regulating diverse biological processes. Further exploration of these mechanisms could enhance our understanding of the therapeutic potential of oxymatrine in treating AS and facilitate the development of oxymatrine-based therapeutic strategies.

The root of the licorice plant (*Glycyrrhiza glabra* Linne) is frequently used as an herbal medicine to treat antinephritis (136). Licorice flavonoids possess antidiabetic, anti-inflammatory, anticancer, and anti-microbial properties (137). He et al. (138) used network pharmacology method to predict the active ingredients of licorice flavonoids with anti-atherosclerotic actions and discovered that Isoliquiritigenin (ISL), a major bioactive component of licorice flavonoids, could protect against vascular endothelial injury induced by TNF by inhibiting the expression of NLRP3, caspase-1 cleavage, and IL-1β production. Moreover, they employed a strategy combined molecular docking and cellular thermal shift assay and demonstrated that ISL could bind to SIRT6 and upregulate SIRT6 expression in TNF-α–stimulated HUVECs. In HUVECs transfected with SIRT6 siRNA, ISL could not achieve its inhibitory role on pyroptosis, whereas NLRP3 inhibition with MCC950 treatment enhanced the inhibitory effect of ISL on pyroptosis. It is noteworthy that the anti-AS effect of ISL was also mediated by the blockade of transient receptor potential channel 5 (TRPC5) channel, a critical regulator of Ca^2+^ signaling, as observed by Qi et al. (139). Their findings demonstrated that ISL administration could attenuate AS lesion and reduce lipid levels in serum of ApoE−/− mice. *In vitro* studies further revealed that ISL blocked VSMC proliferation induced by angiotensin II by regulating TRPC5 activity. However, whether TRPC5 is involved in ISL’s regulation of SIRT6 expression and its potential role in NLRP3 inflammasome activation merits further investigation.

### 4.5 Phosphoinositide 3-kinase/protein kinase B

PI3Ks mediate the phosphorylation of one or more inositol phospholipids at the 3′-position of the inositol ring (140). Multiple cell surface receptors can operate as downstream targets of the PI3K signaling pathway, which is an essential regulator of cell polarization and survival (141). Numerous disorders are linked to excessive PI3K signaling pathway activation or inactivation, emphasizing the significance of appropriate regulation of PI3K (142). A number of AKT subfamilies of AGC serine/threonine kinases, including AKT1, AKT2, and AKT3, appear to be activated consistently in response to PI3K activation (142). Targeting PI3K/AKT signaling may influence the activation of the NLRP3 inflammasome, thus affecting the progression of AS. For instance, He et al. (143) identified a significant bioactive extract from *Radix Bupleuri*, saikosaponin-A (SSA), can suppress ox-LDL–induced pyroptosis, reduce lipoprotein uptake, and prevent the formation of foam cells and the expression of CD36 and density lipoprotein receptor-1 in THP-1 cells. SSA also markedly increased the expression of peroxisome proliferator-activated receptor and ATP-binding cassette transporter A1, as well as cholesterol efflux. Further analysis revealed that SSA prevented the production of pro-inflammatory cytokines (IL-1β, IL-18, IL-6, TNF-α, and MCP-1), NLRP3 inflammasome assembly, and ox-LDL–induced activation of PI3K-AKT and NF-κB signaling in the cells. However, the efficacy of SSA in AS progression has only been assessed in THP-1 macrophages. Therefore, its potential function warrants further evaluated in other cell types implicated in AS pathogenesis. More importantly, the potential physiological role of SSA in AS progression necessitates comprehensive exploration through the use of multiple AS animal models prior to additional pre-clinical evaluations. Regarding the underlying mechanisms, future studies should thoroughly evaluate the relative contribution of the PI3K-AKT pathway compared to other pathways, such as the NF-κB pathway, in the activation of NLRP3 inflammasome and AS development.

Other natural products can also affect the activation of the NLRP3 inflammasome and AS progression by targeting PI3K/AKT pathway. Hydroxysafflor yellow A (HSYA) is a bioactive compound extracted from *Carthamus tinctorius L*. Feng et al. (144) found that HSYA can alleviate plaque formation, lymphangiogenesis, and inflammatory mediators such as IL-6, TNF-α, and Vascular endothelial growth factor C (VEGF-C) in the aorta of ApoE−/− mice. *In vitro* studies revealed that the inhibitory effect was mediated by regulating AKT/mTOR and NF-κB activation through inhibiting PI3K signaling in macrophages. As discussed above, these signaling pathways may influence the activation of the NLRP3 inflammasome, as demonstrated by Xu et al. (145), which revealed that HSYA inhibits NLRP3 activation in mouse RAW264.7 macrophages by binding to xanthine oxidase. Additionally, HSYA has been shown to suppress OGD/R-induced pyroptosis in HUVECs by inhibiting NLRP3 activation (146). Although the signaling pathways triggered by HSYA in HUVECs remain unidentified, it is reasonable to hypothesize that the PI3K-AKT and the NF-κB pathways may be involved. Notably, SSA can activate multiple signaling pathways, including Tumor necrosis factor receptor 1 (TNFR1)/NF-κB, MAPKs, and TLR4/Rac1/AKT, during treatment of AS (147). Determining their relative contributions in various AS-associated cell types warrants further investigation in future studies.

### 4.6 Pyruvate kinase type M2

PKM2 is a pyruvate kinase that catalyzes the generation of ATP and pyruvate and plays a critical role in the regulation of glycolysis under various conditions (148). The PKM2-dependent lactate-dependent PKR phosphorylation is necessary for NLRP3 activation (149). PKM2 positively correlated with AS development by regulating the activation of the inflammasome element PKR (also known as Eukaryotic translation initiation factor 2-alpha kinase 2 (EIF2AK2)) to facilitate inflammasome formation (149, 150). A recent study has demonstrated that PKM2 is the target of salvianolic acid A (SAA) in treating AS. SAA is the primary water-soluble and biologically active component of the root of *Salvia miltiorrhiza* (Danshen) (151), a popular health food and CHM that has been linked to several activities, including the prevention and treatment of cardiovascular and metabolic disorders. To study the role of SAA in AS, Zhu et al. (151) established a diabetic AS model using the ApoE−/− mice, which was treated with streptozocin and a Western diet (WD). Treatment with SAA-ameliorated streptozocin and WD-induced atherosclerotic plaque symptoms decreased the expression of NLRP3, ASC, cleaved-GSDMD, caspase-1, and IL-18 in the endothelial cells of diabetic AS mice. In studies exploring the mechanisms, the researchers discovered that SAA interacts with PKM2 and inhibits phosphorylation at Y105, which suppresses PKM2 nuclear translocation and inhibits lactic acid–dependent PKR phosphorylation and subsequent pyroptosis of endothelial cells. The anti-AS effect of SAA has also been demonstrated by Ma et al. (152) using vitamin D3–injected Zucker Diabetic Fatty (ZDF) rats treated with a HFD. It is interesting to find that the NLRP3 inflammasome activation in ZDF rats was also inhibited by administration of SAA. It seems likely that the AS model induced in ZDF rats was much susceptible to SAA compared to that induced in ApoE−/− mice, because the drug dose used in ZDF rats (0.5 and 1 mg/kg/day) was remarkably lower than that used in ApoE−/− mice (10 and 20 mg/kg/day). Extensive pharmacodynamics analysis should be performed to evaluate the potential therapeutic efficacy and safety of SAA in both animal species. Additionally, it is crucial to investigate the underlying mechanisms by which SAA regulates PKM2 activity and expression in the AS model of ZDF rats. These studies may involve exploring the interaction between SAA and PKM2 and determining the signaling pathways that are activated or inhibited by SAA.

### 4.7 MicroRNAs

MicroRNAs (miRNAs) are a class of endogenous, evolutionarily conserved, short non-coding RNA sequences, playing an essential role in controlling the progression of pyroptosis by targeting miRNAs (153, 154). Zhilong Huoxue Tongyu Capsule (ZHTC) is a traditional Chinese remedy widely used in the treatment of cardiovascular and cerebrovascular diseases (155) and hypertension (156). Liu et al. (157) showed that ZHTC can reduce the levels of NLRP3, ASC, caspase-1, IL-1β, and IL-18 and alleviate the pyroptosis of human coronary artery endothelial cells (HCAECs) after exposure to ox-LDL. Moreover, ZHTC could enhance the expression of miR-30b-5p in HCAECs, which binds to NLRP3 and negatively regulates with NLRP3 activation. Using miR-30b-5p mimic and inhibitor, the researchers demonstrated that miR-30b-5p serves as a key mediator induced by ZHTC to exert its anti-NLRP3 inflammasome and anti-AS effects. However, the efficacy of ZHTC in AS requires further evaluation by other research groups. The precise ingredients of ZHTC that promote the expression miR-30b-5p expression remain unidentified. Determining the interactions between bioactive ingredients of ZHTC and miR-30b-5p or the NLRP3 inflammasome may deepen our understanding of the regulatory mechanisms by which ZHTC modulates AS progression. Additionally, elucidating the downstream targets of miR-30b-5p that are modulated by ZHTC could provide insights into novel therapeutic pathways for AS. Investigating the effects of ZHTC on other cell types implicated in AS pathogenesis may reveal additional mechanisms by which ZHTC modulates AS progression. Elucidating these issues might contribute to the development of more effective treatment strategies for AS based on ZHTC.

As discussed above, Paeonol is another compound that can affect NLRP3 inflammasome activation and AS progression by regulating an miRNA. As a major bioactive component of Cortex Moutan (158, 159), Paeonol can reduce the expression levels of NLRP3, ASC, caspase-1, ICAM-1, IL-1β, and IL-6 to alleviate HFD-induced AS (84). In this process, its function in enhancing the expression of miR-223 plays a critical role. The upregulation of plasma exosomal miR-223 could inhibit endothelial cell-mediated inflammation. However, its roles in regulating Neutrophil extracellular traps (NET)-mediated foam cell inflammation (83) and suppressing the generation of HIBA by the gut microflora (82) both contribute Paeonol’s anti-AS effects. The interactions between CitH3 and ROS/TXNIP pathways in regulating the activation of NLRP3 inflammasome warrant further investigation.

### 4.8 The gut microflora and associated metabolites

Gut microbiota plays a crucial role in the development of AS, with an imbalance in the Firmicutes/Bacteroidetes ratio closely associated with metabolic disorders (160). Microbial metabolites such as TMAO can induce oxidative stress, inhibit Endothelial Nitric Oxide Synthase (eNOS) activity, and reduce NO production, thereby exacerbating endothelial dysfunction (161–163). Additionally, metabolites like aminoadipic acid (AAA) may regulate NLRP3 activation, further aggravating inflammation and promoting AS progression (164). The probiotic *E. coli* Nissle 1917 (EcN) may alleviate AS by inhibiting NLRP3 activation through the modulation of gut microbiota and serum metabolites, offering a potential therapeutic strategy (165). Combined CHM improves lipid accumulation and metabolic disorders induced by a HFD via regulating the microbiota-gut-liver axis and metabolic pathways, showing potential as a therapeutic strategy for obesity (166). Therefore, it is possible for future research to explore the use of probiotics as an additional component in the treatment of AS with current medications. Recent studies have linked unbalanced gut microflora to various ailments, including AS (167). The gut microflora is also found to be a regulating target of several natural compounds and traditional medicines in treating AS. An example of this line of evidence was provided by Qi et al. (64), showing that the structure and composition of rabbit intestinal flora can be significantly altered by TXL as mentioned above. TXL could attenuate high cholesterol diet and balloon injury–induced imbalance between Firmicutes and Bacteroidetes in New Zealand white rabbits. Meanwhile, TXL treatment also changed the levels of transferulic acid. These changes may have an impact on the expression of NLRP3 and its downstream inflammatory cytokines. Although TXL could induce changes of gut microbiota in an AS-related rabbit model, the specific bacterial species and metabolites that exert anti-AS effects in other species of animal models such as ApoE−/− mice remain further identification. The functions of these bacterial species also require evaluation in both animal and cellular models of AS. Extensive investigation of the interactions between microbial metabolites and the components of the NLRP3 inflammasome can further enhance our understanding of mechanisms by which TXL regulates AS progression.

Another formula that affects AS by regulating microbiota and associated metabolic pathways is the Tangzhiqing formula (TZQ), a traditional medicine widely used to treat glucose and lipid metabolism. TZQ could reduce the diameter of the common carotid artery and blood flow velocity, blood levels of lipids, and inflammatory cytokines by inhibiting NLRP3 inflammasome–mediated pyroptosis in HFD-treated ApoE−/− mice (168). Using metabolomics, the researchers found that TZQ regulated the metabolism of arachidonic acid, glycerophospholipids, steroid hormones, and unsaturated fatty acids by modulating P450 and COX-2. However, the link between the altered metabolic pathways and NLRP3 inflammasome activation has not yet been determined. Moreover, the efficacy of TZQ in AS should be confirmed by other research groups in multiple cellular and animal models of AS. Additionally, the identification of the bioactive ingredients of TZQ that have anti-AS effects may notably enhance our understanding of the molecular mechanisms of TZQ to ameliorate AS-related symptoms.

Natural products can affect AS progression by regulating gut microbiota and NLRP3 inflammasome. Ma et al. (169) reported that NR1 could reduce AS-associated conditions in HFD-induced AS rats. Fecal analysis disclosed the changes in the composition of pathogenic and probiotic bacteria, such as the decline of Firmicutes and Proteobacteria and the increase of Bacteroidetes. Meanwhile, NR1 also inhibited the level of plasma NO and eNOS in plasma of rats and thus suppressed the activation of the NLRP3 inflammasome. While the effect of NR1 has been extensively demonstrated by many other researcher groups, various mechanisms have been identified (170, 171). Whether the gut microbiota and NLRP3 inflammasome have correlations remains undetermined. Identification of the altered metabolites triggered by NR1 can significantly enhance our knowledge of the regulatory mechanisms by which NR1 ameliorates AS progression. AAA is a harmful metabolite generated during progression of AS. Its level in serum of AS patients is much higher than that in heathy individuals (164). AAA supplementation could enhance aortic plaque formation and increase inflammatory cytokines in serum and Malondialdehyde (MDA) and SOD levels in liver of AS mice. AAA exposure could enhance ROS production and thus induce production of ASC, TXNIP, NLPR3, and caspase-1 in HUVECs. Therefore, various CHMs and natural compounds can influence AS progression by regulating the gut microbiota and associated metabolites, which affects the activation of NLRP3 inflammasome in multiple cell types implicated in AS pathogenesis. Future studies may focus on identifying of the bioactive metabolites of NR1 and other potential natural compounds. Additionally, it is noteworthy to investigate the correlations and interplays between the bioactive compounds and the gut microbiota, as well as the underlying molecular mechanisms by which the natural compounds regulates AS progression.

## 5 Conclusions and limitations

**Figure 4:**
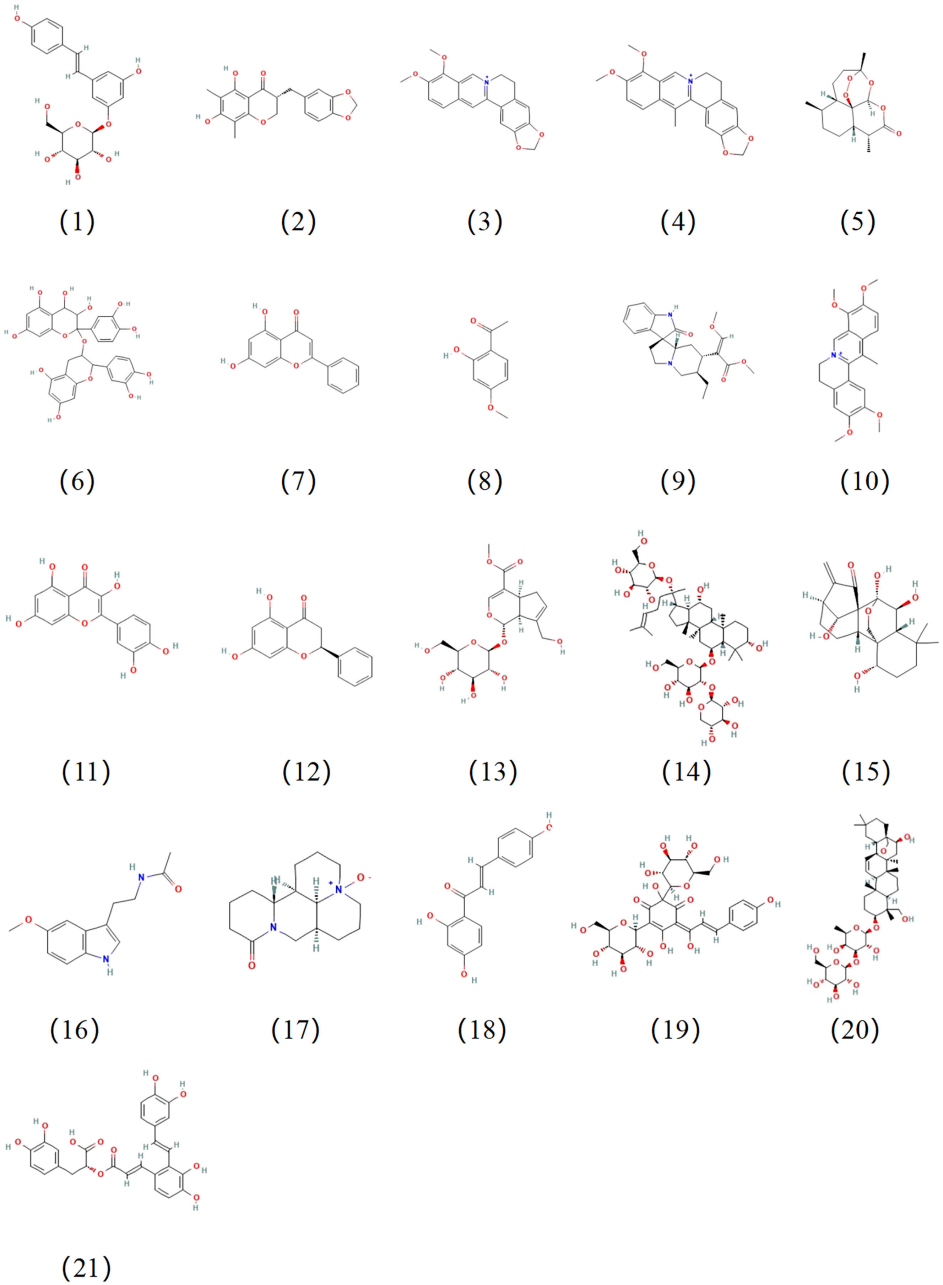
Chemical structures of the natural compounds suppress the progression of AS by regulating NLRP3 activation. The compound numbers correspond to the compound names in **Table 2**.

This study offers a summary of the principal findings from recent studies in the literature and elucidates how CHMs and CHM-derived natural compounds (chemical structures are shown in **Figure 4**) regulate NLRP3 inflammasome activity and thereby influence AS progression in animal and cellular models. The critical review of these studies indicated that certain CHMs or natural compounds can alleviate AS-associated pathologies by inhibiting the NLRP3 inflammasome through a variety of regulatory mechanisms. Consequently, the appropriate application of CHMs or natural compounds may represent a promising approach for treating AS through modulating the activation of the NLRP3 inflammasome. However, the subsequent clinical application of these drugs in practice may present challenges. In this section, we will discuss several limitations of current research related to NLRP3 inflammasome in treating AS and provide corresponding solutions based on our knowledge and understanding.

The unstandardized experimental design may limit the reproducibility of the results. Firstly, the aforementioned studies may not have clearly defined the specific disease stages of AS targeted by CHM interventions (e.g., early plaque formation stage vs. late plaque rupture stage). The lack of clarity may affect the relevance and comparability of experimental results. Secondly, differences in administration routes (e.g., oral or injection), dosing frequency, and duration may significantly influence the bioavailability and efficacy of the drugs. Additionally, the wide variation in dosage ranges used for the same drug across different studies further contributes to inconsistent results. To reduce such heterogeneity and improve the reproducibility of results, it is recommended that future studies adopt standardized experimental designs, such as unified animal models, dosage ranges, or detection methods. The current experimental models for evaluating CHM efficacy in AS have notable limitations. Mouse models naturally lack cholesterol ester transfer protein (CETP), resulting in cholesterol and lipoprotein metabolism that differs from humans (172–174). Even with transgenic modifications to induce hypercholesterolemia, AS pathogenesis and cardiovascular anatomy still exhibit significant differences from humans (172–174). The most commonly used mouse model for AS is the ApoE−/− mouse (175). However, the lipid metabolism mechanism of ApoE−/− mice differs significantly from that of humans, with disease progression being driven by VLDL rather than LDL and abnormally high HDL levels (176, 177). As a result of these metabolic differences, the clinical translation of CHM research findings in these models may be limited, highlighting the need for more representative models such as humanized animals to bridge the gap between preclinical and clinical studies. Additionally, *in vitro* models of AS play a crucial role in studying its mechanisms and screening potential drugs. However, they also have significant limitations, including the use of a single cell type, the lack of a dynamic blood flow environment, difficulty in simulating complex plaque structures, and the inability to reflect systemic metabolic effects (178). To overcome these limitations, future research can integrate multicellular co-culture systems, 3D models, microfluidic chips, and organoid technology to better mimic the pathophysiological conditions of AS.

Over 12,000 different medicines, including plants, animals, and minerals, and approximately 100,000 prescriptions have been used in CHMs (179, 180). All of these medications have intricate chemical components, some of which are typically recognized by the human body as dangerous substances that invade the human body (181). Different chemical components undergo complex reactions in the human body, including cross-reactions (182–184). Thus, the identification of active ingredients is a challenge for the development of specific regulators of the NLRP3 inflammasome that may have an inhibitory effect on AS-associated symptoms. Most herbal-derived chemicals exhibit extremely low bioavailability due to rapid metabolism, poor absorption, or low water solubility. For example, resveratrol and curcumin both show oral bioavailability below 1% (179, 185, 186), whereas TSIIA achieves less than 3.5% (187). Various strategies have been developed to improve the bioavailability of drugs. Liposomes, based on phospholipid vesicle systems, are one of the most widely studied lipid drug carriers due to their amphiphilicity, high biocompatibility, and strong affinity for biological membranes, which allow for rapid absorption through endocytosis or enhanced diffusion across lipophilic cell membranes (188–190). Meriva^®^ (a curcumin-phosphatidylcholine complex) has shown in clinical trials that, compared to unformulated curcumin, it increases curcumin bioavailability and demonstrates higher adherence to treatment with fewer adverse events (188–191). Additionally, TSIIA oral bioavailability is enhanced by encapsulating it into lipid nanocapsules (LNCs) (192). Moreover, many other approaches such as microspheres have proven effective in delivering drugs to tissues and enhancing the bioavailability of various CHMs (193). The carrier systems for a particular drug need to be determined according to the features of the drug, and their efficacy needs to be evaluated in extensive *in vivo* studies. Thus, advanced drug delivery systems are crucial to improve their bioavailability and therapeutic efficacy.

The efficacy of the CHMs and natural compounds in AS has been extensively investigated in recent studies. Despite their long history of use, many CHM-derived compounds have not been sufficiently studied for safety, particularly in terms of long-term use and potential adverse effects. These compounds may exhibit dose-dependent toxicity (194), toxicity of metabolites (195), or off-target effects (196, 197) that are not immediately apparent in preclinical models. Furthermore, the lack of standardization in production and formulation can lead to variability in the quality and potency of CHM products, making it challenging to consistently assess their safety and efficacy (198). In the present study, we found that only 17 out of 38 investigations provided *in vivo* toxicity data for the AS models. To address the potential toxicity of CHMs and natural herbal compounds, extensive toxicity evaluation should be conducted using multiple animal models in pre-clinical tests. The toxicity analysis should be listed as a standard procedure in efficacy studies of CHMs or natural compounds. The related molecular mechanisms need to be further investigated, and the resultant evidence may be helpful in reducing the toxicity of drugs or developing alternative drugs for treating AS. It is also important to include other drugs to reduce the side effects observed during disease treatment based on pharmacological evidence of different drugs. A question that may influence the toxicity of the drugs is their quality, as the proportion of the bioactive ingredients in a particular herbal medicine can be affected by various factors, such as increasing air and soil pollution, pesticides, heavy materials, and the growing environment. Thus, it is important to promote the application of new strategies, combine network pharmacology and systems biology, identify and determine quality markers, and thus decrease the variation of herbal plants and standardize the procedures of producing CHMs (199). To address this issue, it is necessary for governments, research institutions, the industry, and consumers to work together to establish a global monitoring system and to adopt advanced technologies to ensure the quality of CHMs (200). By implementing standardized preparation processes, including unified quality control standards, standardized extraction techniques, and rigorous quality testing methods, the variability of bioactive components can be effectively reduced, ensuring the consistency and stability of each batch of herbal products. This not only helps improve the clinical efficacy of CHM but also enhances its recognition and competitiveness in the international market.

The unclear molecular mechanism of certain CHMs or natural compounds in targeting NLRP3 inflammasome and treating AS is another issue that is associates with toxicity of drugs and limits the clinical application of anti-AS CHMs. High performance liquid chromatography-tandem mass spectrometry (HPLC-MS)–based strategies can identify numerous compounds in a particular CHM, and subsequent functional verification of certain compounds in disease progression must be conducted using methods that combined component purification and pharmacological analyses. Numerous methods and strategies have been developed for this purpose. For instance, the application of Chinmedomics—a strategy combining systems biology, serum pharmacochemistry, and bioinformatics to evaluate the efficacy of CHMs (201)—may largely promote the identification of effective components of a particular medicine and the associated molecules and signaling pathways in treating AS. Furthermore, with the continuous development of the strategy combined with network pharmacology analysis and the Clustered Regularly Interspaced Short Palindromic Repeats (CRISPR) technology, we can predict the potential binding partners of CHM ingredients and make genetic deletion of these binding partners or regulators of the NLRP3 inflammasome to elucidate the molecular mechanisms of CHMs in modulating NLRP3 inflammasome activation and AS progression. Single-cell RNA sequencing (scRNA-seq) and multi-omics approaches can further reveal the dynamic changes of different immune cell subsets in inflammatory responses, providing more precise molecular targets for personalized therapeutic strategies for AS treatment (202). These advanced technologies offer promising therapeutic potential for AS by targeting NLRP3 inflammasome or its regulators and enabling precise personalized treatments of the disease.

## 6 Trends and future directions

As discussed above, numerous CHMs and CHM-derived natural compounds can ameliorate AS progression by modulating the activation of the NLRP3 inflammasome through a variety of regulatory mechanisms. These studies offer a promising approach for AS treatment, but a few challenges require to be solved before their clinical applications. Therefore, future studies should overcome the major challenges of current studies: (1) To reduce the heterogeneity that widely occurs in current experimental design and to improve the reproducibility of results, future studies should adopt standardized experimental designs, such as unified animal models, dosage ranges, or detection methods. (2) To overcome the differences of animal and cellular models of AS with real human disease, future research can employ humanized animals and integrate multicellular co-culture systems, 3D models, microfluidic chips, and organoid technology to better mimic the pathophysiological conditions of AS. (3) The issue of low bioavailability can be improved by the application of carrier systems such as liposomes, Meriva^®^, nanoparticles, and microspheres. (4) Extensive mechanistic studies can help clarify the molecular targets of CHMs and natural compounds and, therefore, improve the drug specificity and reduce toxicity. (5) Single-cell sequencing and multi-omics strategies can be used in identification of CHM ingredients and metabolites with anti-NLRP3 inflammasome and anti-AS activity and the cell types highly sensitive to the active components of the drugs and, therefore, can facilitate the establishment of personalized CHM therapies. (6) CRISPR-based approaches can be used in modifying the identified drug targets or regulators of the NLRP3 inflammasome in AS and facilitating the functional study of genes. (7) The applications of Chinmedomics analysis can be helpful in establishing quality markers for CHMs and ensuring the standardization and consistency of CHM products.

Based on our understanding of the current research progression of the field, we propose a few questions that may deepen the mechanistic studies and facilitate the development of novel anti-AS drugs. (1) What is the effect of CHMs and natural compounds on the non-canonical pathway of the NLRP3 inflammasome activation in AS progression? (2) Can we combine certain CHMs or natural compounds with immune checkpoint inhibitors to modulate the progression of AS? (3) What role do long non-coding RNAs play in CHM- and natural compounds-mediated NLRP3 suppression and AS progression? (4) What is the association between CHM-induced miRNAs such as miR-223 and NLRP3 inflammasome regulators such as CitH3, PKM2, and SIRTs in AS progression? (5) What microbial species or metabolites play the inhibitory roles on AS? (6) Can the anti-inflammasome and anti-AS effects of CHMs and natural compounds be transferred by fecal transplantation? Answers to these questions may lead to novel therapeutic strategies for AS treatment. Overall, continued investigation in these areas is essential for advancing our understanding of AS pathogenesis and developing more effective therapies.

## Glossary

AAA: Aminoadipic acid
ABCA1: ATP-binding cassette transporter A1
ABCG1: ATP-binding cassette transporter G1
AGEs: Advanced glycation end products
AKT: Protein kinase B
AS: Atherosclerosis
ASC: Apoptosis-associated speck-like protein; containing a caspase recruitment domain
ASCVD: Arteriosclerotic cardiovascular disease
AT: Antithrombin III
CARD: C-terminal caspase-recruitment domain
CCL2: (Chemokine) C-C motif: ligand 2
CETP: Cholesterol ester transfer protein
CHM: Chinese herbal medicines
CLS1: Cardiolipin synthetase 1
DAMPs: Damage-associated molecular patterns
DHC: Isorhynchophylline
IRN: dehydrocorydaline
EcN: E. coli Nissle 1917
GN combination: Geniposide combined with notoginsenoside R1
GP: Geniposide
GSDMD: N-terminal portion of gasdermin D
HCAECs: Human coronary artery endothelial cells
HFD: High-fat diet
HMG-CoA: 3-Hydroxy-3-methylglutaryl–coenzyme A
HO-1: Heme oxygenase-1
HUVECs: Human umbilical vein endothelial cells
IL-6: Interleukin-6
ISL: Isoliquiritigenin
LC3I: Cytoplasmic LC3
LC3II: Autophagosome membrane-type LC3
LDH: Lactate dehydrogenase
LDL-C: Low-density lipoprotein cholesterol
lncRNAs: Long non-coding RNAs
LNCs: lipid nanocapsules
LOX-1: Lectin-like oxidized low-density lipoprotein (LDL) receptor-1
LRR: C-terminal leucine-rich repeat
MCP-1: Monocyte chemotactic protein-1
MMPs: Matrix metalloproteinases
mtDNA: Mitochondrial DNA
mTOR: Mammalian target of rapamycin
MyD88: Myeloid differentiation factor 88
NF-κB: Nuclear factor kappa B
NLRs: NOD-like receptors
NR: Notoginsenoside R1
NR1: notoginsenoside R1
NRF2: Nuclear factor E2–related factor 2
P2X7R: Purinergic 2X7 receptor
Pae: Paeonol
PAMPs: Pathogen-associated molecular patterns
PI3K: Phosphoinositide 3-kinase
PKM2: Pyruvate kinase type M2
PYD: N-terminal pyrin domain
QRHX: Qingre Huoxue Decoction
ROS: Reactive oxygen species
scRNA-seq: Single-cell RNA sequencing;
SIRT: Sirtuins
SNPs: Single-nucleotide polymorphisms
STZ: Streptozotocin
TC: Total cholesterol
TLR4: Toll-like receptor 4
TLRs: Toll-like receptors
TNF: Tumor necrosis factor
TSIIA: Tanshinone IIA
TXL: Tongxinluo
TXNIP: Thioredoxin interacting protein
TZQ: Tanghiqing formula
VSMCs: Vascular smooth muscle cells
WD: Western diet
ZHTC: Zhilong Huoxue Tongyu Capsule
